# Composites of Polylactic Acid with Diatomaceous Earth for 3D-Printing Biocompatible Scaffolds: A Systematic Study of Their Mechanical, Thermal, and Biocompatibility Properties

**DOI:** 10.3390/bioengineering11111059

**Published:** 2024-10-24

**Authors:** Lilliam Trejos-Soto, Gabriel O. Rivas-Hernández, Rodrigo Mora-Bolaños, Nathalia Vargas-Valverde, Abraham Valerio, Andrea Ulloa-Fernández, Jorge Oviedo-Quirós, Alfonso García-Piñeres, Sergio A. Paniagua, Carolina Centeno-Cerdas, Leonardo Lesser-Rojas

**Affiliations:** 1 Master Program of Engineering in Medical Devices, School of Materials Science and Engineering, Tecnológico de Costa Rica, Cartago 30101, Costa Rica; 2 Biotechnology Research Center (CIB), Biology School, Tecnológico de Costa Rica, Cartago 30101, Costa Ricaccenteno@itcr.ac.cr (C.C.-C.); 3 Bioengineering Department, Universidad Carlos III de Madrid, 28911 Leganés, Madrid, Spain; 4 National Nanotechnology Laboratory (LANOTEC), National Center for High Technology (CENAT), San José 1174, Costa Ricaspaniagua@cenat.ac.cr (S.A.P.); 5 Advanced Materials Science and Engineering Master Programme (AMASE), Université de Lorraine, 54000 Nancy, France; 6 Faculty of Chemistry and Biology, Université Grenoble Alpes, 38400 Saint Martin d’Hères, France; 7 School of Physics, Universidad de Costa Rica, San José 11501, Costa Rica; 8 Advanced Materials and Liquid Crystal Institute & Materials Science Graduate Program, Kent State University, Kent, OH 44242, USA; 9 Craniomaxillofacial Cleft Palate Unit, National Children’s Hospital “Dr. Carlos Sáenz Herrera”, San José 10103, Costa Rica; 10 Faculty of Dentistry, Universidad de Costa Rica, San José 11501, Costa Rica; 11 Cellular and Molecular Biology Research Center (CIBCM), Universidad de Costa Rica, San José 11501, Costa Rica; 12 Department of Biochemistry, School of Medicine, Universidad de Costa Rica, San José 11501, Costa Rica; 13 Research Center in Atomic, Nuclear and Molecular Sciences (CICANUM), Universidad de Costa Rica, San José 11501, Costa Rica

**Keywords:** biodegradable, biomaterial, polylactic acid (PLA), silica-filled composite, gamma sterilization, osteoconduction

## Abstract

This study explores the development of biocompatible scaffolds for bone regeneration, utilizing polylactic acid (PLA) combined with calcium phosphate as a pH buffer and diatomaceous earth as a biocompatibilizer. These materials were extruded and 3D-printed to enhance cell adhesion and biodegradability after enough cell growth. The biocompatibility of the resulting composites, with different proportions of the components and sterilization methods, was tested according to the ISO 10993 protocol. The optimal performance, with nearly zero cytotoxicity, was observed with 20 PLA/1 CP/1 DE mass ratios and gamma sterilization. Tension analysis and scanning electron microscopy (SEM) were applied to the 3D-printed composites, which were also analyzed by differential scanning calorimetry (DSC) to understand the origin of the tension properties better, which were comparable to those of cancellous bone. Degradation tests under physiological conditions for 13 weeks showed no significant mass loss. Furthermore, it was observed that cell adhesion, viability, proliferation, and osteoconduction are possible in the scaffolds studied, opening opportunities for future studies to substantiate the use of 3D-printed silica-filled composites as an alternative to homologous implants for various bone regeneration applications.

## 1. Introduction

The biomedical industry has been in search of biocompatible scaffolding for promoting regeneration as an alternative to autologous tissue grafting [1], which has the complication of morbidity in the excision site. An important application of biocompatible scaffolding is the repair of a cleft palate, in which the two plates of the skull that form the hard palate and the soft palate fail to join. This leads to difficulties in feeding and speaking, with a concomitant high frequency of infections. When the cleft extends into the maxillary alveolar ridge, the usual treatment is inserting bone tissue from the individual’s iliac crest region of the pelvic bone during childhood [2], which involves the comorbidity associated with two incisions [3]. Hence, there is an evident need for new biocompatible materials that promote a patient’s own osteoblasts’ growth, with mechanical properties and fitting geometry similar to that of bone.

PLA is a common polymer used in the biomedical implant industry due to its rigidity (elastic modulus up to 2.7 GPa) and appropriate glass transition temperature (60–65 °C) [4], as well as its biodegradability and minimal toxicity [5]. However, PLA displays low cell adhesion due to its hydrophobic nature [6]. Nevertheless, polymers such as PLA can be combined with other chemicals to improve properties essential for osseointegration.

A major advantage of composites is the possibility to fine-tune and optimize basic properties such as their elasticity, strength, and thermal stability [7], as well as stimulating cell adhesion, growth and proliferation, and polymer degradation and absorption. Exposing polymeric biomaterials to inorganic chemicals can significantly enhance cell–material interactions [8,9]. Combining polymers with inorganic materials and bioactive chemicals enables customization of the pore shape and size for improved cell mobility while reducing inflammatory responses, controlling degradation rates, and improving biocompatibility [10,11,12].

Besides traditional materials like calcium phosphates, hydroxyapatite, carboxyhydroxyapatite, and calcium oxides, research on incorporating inorganic materials to enhance the biocompatibility and biodegradability of polymeric matrices is still very limited [13,14,15]. There are few reports on using inorganic fillers to increase cell proliferation; however, those that use calcium phosphate derivatives seem promising [13,16]. Recently, silica-containing fillers have gained attention for their incorporation into polymeric composites [17].

Silicon and silica provide multiple benefits for bone scaffolds and implants. Silicon promotes osteoblast proliferation, differentiation, and collagen production, possibly through the stimulation of the prolyl hydroxylase enzyme involved in collagen synthesis [18,19]. Similar effects have been observed in materials with a high silica content, such as Bioglass 45S5^®^ [20]. Silicon also promotes biomimetic precipitation by increasing solubility through defects in the material’s crystal structure, reducing the grain size, and generating a negative surface charge, which further improves osteoblast adhesion and collagen formation [21,22,23,24,25]. Silica-containing materials undergo partial dissolution, forming an amorphous silicon layer that increases type I collagen synthesis, apatite formation, and osteoblast proliferation and differentiation [26]. This process enhances extracellular matrix formation, biomineralization, tissue regeneration, and bone remodeling [27].

Recent efforts have focused on integrating silica-based micro- and nanostructured compounds into polymeric materials for biomedical applications, improving cell proliferation in 3D-printed scaffolds [16,17]. In bone tissue regeneration, the substitution of hydroxyapatite with silicon has promoted mature bone formation and orderly collagen fibrils [28]. Composites with a high silicon content (1:1) increased osteocalcin mRNA and osteopontin expression, while a lower silicon content enhanced osteoblast differentiation [29]. Doping tricalcium phosphate scaffolds with silica and zinc oxide increased their compressive strength and fetal osteoblast proliferation in vitro [16]. Silica has been used in insulin–hydrogel injections, boosting alkaline phosphatase (ALP) activity and matrix formation in osteosarcoma cells [30]. Silica–calcium phosphate nanocomposites seeded with bone marrow stromal cells promoted cell proliferation and upregulated osteogenic markers like Runt-Related Transcription Factor 2 (RUNX2), ALP, and collagen I [31]. Additionally, incorporating silica nanoparticles into electrospun carbon nanofibers improved the hydrophilicity and osteosarcoma cells proliferation in the scaffold [32]. Mesoporous silica nanoparticles also exhibit osteogenic and angiogenic properties [33].

It is necessary to understand the mechanical properties of these composites for the optimal production and performance of bone regeneration scaffolds. With the addition of particulate fillers, the mechanical properties are expected to improve, especially if the filler–polymer matrix adhesion enables load transfer from the matrix and if the fillers are able to prevent crack propagation through the composite. The most critical issue in composite processing is to optimize the dispersion and distribution of the fillers to achieve a homogeneous product [8]. Furthermore, these composites can be 3D-printed by fused deposition modeling (FDM) with nozzle-based dispensing systems. Nozzle-deposition-based techniques allow 3D structures and complex geometry models to be built with precise control and reproducibility, using a large variety of materials, with sterilization during the process, and with high biocompatibility and dimensional stability. Stereolithography (SLA) 3D printing is an alternative, but customization of light-curable resins is a more complex endeavor than extruding composite filaments. Also, SLA resins usually display a fast degradation rate, and therefore, mediocre mechanical properties for this purpose [34,35,36].

The polymer composition determines the nature of the porous structure of the scaffolds (pore size, shape, and interconnectivity). The porosity of the scaffold enables and facilitates cell migration, ingrowth, and effective nutrient distribution, as well as waste removal. Therefore, an appropriate scaffold porosity is essential for successful tissue engineering [37]. To facilitate the formation of new tissue using scaffolds, their structure should have a highly interconnected pore network to allow cell growth and mass transport, as well as degradation at a pace that allows their replacement by nascent tissue. Ideally, they should also exhibit a surface chemistry suitable for cell attachment, proliferation, and differentiation [38].

We hypothesize that biocompatible and biodegradable scaffolds for bone regeneration, based on a composite material made from polylactic acid (PLA) combined with calcium phosphate as a pH buffer and diatomaceous earth (DE) as a biocompatibilizer, can be effectively extruded and 3D-printed to enhance cell adhesion and biodegradability after enough cell growth. DE is composed of fossilized remains of diatoms, a type of hard-shelled protist. DE’s chemical composition is silica in its majority, and the particle size is typically a few micrometers in any direction [39].

In this work, polymeric composites with different percentages of additives were prepared via casting and then cut and extruded to 3D-print probes used in dynamic mechanical analysis, as well as porous scaffolds based on computerized axial tomography of trabecular bone. A battery of characterizations was performed to evaluate the mechanical properties of the different compositions. Additionally, we assessed the biocompatibility of the composites based on their chemical composition, the sterilization method, and the scaffold pore size.

## 2. Materials and Methods

### 2.1. Materials and Generation of Composites

Extrusion-grade PLA, calcium phosphate (CP), diatomaceous earth (DE), and laboratory-grade chloroform were used for the generation of the composites. The material properties and a summary of the suppliers are displayed in Table 1. Table 2 summarizes the composites and their mass ratios.

Supplementary Material Figure S1 shows SEM images of the DE used. DE was incorporated into the PLA at 20 PLA/1 DE and 20 PLA/5 DE ratios to assess the influence of DE at low and high loadings, respectively, with a 20 PLA/1 CP ratio in both cases to buffer any acidity from the PLA terminal groups. For the low DE loading, a composite with no CP was also prepared. A 1-to-10 ratio m/V was maintained between the PLA and chloroform. The mixture was typically stirred for 4 h at near-boiling temperature in a chemical hood, and once the PLA dispersed, the mixtures were decanted into a large but shallow glass container and left to dry in the chemical hood. Once the chloroform odor subsided, the dried cast was cut into square pellets of about 0.5 cm^2^.

Pellets were fed into a Filabot EX2 extruder with a 1.75 mm nozzle, and filaments were produced at 175 °C while varying the extrusion rate to ensure the most homogeneous filaments possible, resulting in an average diameter of 1.75 ± 0.15 mm. 

### 2.2. Viability and Cytotoxicity Assays by Extracts after Sterilization Procedures

Cytotoxicity assays were performed as indicated in the ISO 10993 protocol [40]. The cell line used for these experiments was the MC3T3-E1 Subclone 4 CRL-2593™, purchased from the ATCC (Manassas, VA, USA) and maintained in Alpha Minimum Essential Medium with ribonucleosides, deoxyribonucleosides (α-MEM), 2 mM L-glutamine, and 1 mM sodium pyruvate but without ascorbic acid (GIBCO, Catalog No. A1049001) and supplemented with 10% fetal bovine serum (GIBCO/Thermo Fisher Scientific, Waltham, MA, USA) as the cell growth medium for these experiments. 

The materials or composites (PLA, 20PLA/1CP/1DE, 20PLA/1CP/0DE, 20PLA/0CP/1DE and 20PLA/1CP/5DE mass ratios) were sterilized by three different methods as seen in Table 3.

To make the extracts, 0.2 g of each composite was incubated in 1 mL of α-MEM at 37 °C for 24 h.

The MC3T3-E1 cells were seeded at 1 × 10^5^ cells/mL into 96-well plates and incubated at 37 °C and 5% CO_2_ for 24 h. After the incubation, the medium was replaced with the extracts of the composites. The plates were incubated for another 24 h. Viability and cytotoxicity were determined by MTT assay (Sigma-Aldrich, St. Louis, MO) and the cytotoxicity detection kit LDH (Roche^®^/Merck, Darmstadt, Germany), according to the respective manufacturers. The MTT test shows viability through reducing the capacity of living cells, while the quantification of the LDH enzyme released is an indication of cell stress or of a cytotoxic response.

### 2.3. Three-Dimensional Printing and Characterization of the Probes

Filament sections with a diameter of ~1.75 mm were used. A Prusa i3 3D printer (Prusa Research, Prague, Czech Republic) was employed at 195 °C with a hot bed at 60 °C. The probes were designed to be 30.0 mm long, 1.5 mm thick, and 7.0 mm wide with a 15.0 mm long and 3.0 mm wide slit in the middle (see Supplementary Materials Figure S2). The 3D-printed composites were sterilized by gamma rays, which was determined to be the method of sterilization most appropriate for these composites (as discussed in Section 3.1).

#### 2.3.1. Differential Scanning Calorimetry (DSC)

A Q200 differential scanning calorimeter from TA Instruments was used. The samples were encapsulated in an aluminum pan and heated at a rate of 10 °C/min from 25 to 200 °C under a nitrogen atmosphere to determine the glass transition temperature (T_g_) and the curing temperature (T_c_).

#### 2.3.2. Scanning Electron Microscopy (SEM)

The 3D-printed samples were cryo-fractured for cross-section analysis, and the fractured site was mounted face up onto carbon tape, while another section was mounted sideways on the carbon tape for a side view. These were sputtered with gold using a Denton Vacuum Desk V sputter system (Ver 2.1.7.27) at 20 mA for 300 s. The diatomaceous earth was mounted onto silver instead of carbon tape. In all cases, images were taken using a JSM-6390LV (JEOL, Tokyo, Japan) scanning electron microscope with an accelerating voltage of 10–15 kV under a high vacuum. Energy-dispersive X-rays (EDXs) were measured with a liquid-nitrogen-cooled Inca X-sight Si detector (Oxford Instruments, Abingdon, UK). The EDX data were analyzed with Inca Suite version 4.08.

#### 2.3.3. Tension Tests

A Discovery HR-3 rheometer from TA Instruments was employed. For the tension tests, the 3D-printed samples per Section 2.4 were stretched at a ratio of 15 μm/min, and the axial force vs. displacement values were registered, with a maximum of 50 N of tension. Young’s modulus (E) was calculated from Hooke’s Law, where elastic stress ($\sigma_{y}$) is directly proportional to the strain (ϵ), as shown in Equation (1). Elongation strain ϵ was determined from the ratio of the deformation (*δ*) to the length (*L*).

(1) $$E=\frac{\sigma_{y}}{\epsilon }$$

(2) $$\epsilon =\frac{\delta }{L}$$

#### 2.3.4. Degradation Tests under Physiological Conditions

The 3D-printed samples for use in the tension tests were dried overnight in an oven at 40 °C, left to cool, and weighed. They were then placed in a phosphate buffer aqueous solution at a pH of 7.4 and 37 °C (at a 20:1 solution:probe weight ratio) to simulate physiological conditions, with stirring at 120 rpm. The samples were retrieved at timed intervals, rinsed, and dried before weighing them to determine whether mass loss had occurred. The pH of the buffered solution was measured with a Sartorius PB-11 pH-meter (Sartorius, Gottingen, Germany).

### 2.4. The 3D Printing of the Scaffolds

The 20PLA/1CP/1DE composite, sterilized by gamma radiation, was used for the scaffolding tests (after achieving the best results in the viability and cytotoxicity tests, as discussed in Section 3.1). To determine whether the average size of the pores influences the proliferation of MC3T3-E1 cells, the scaffolds were printed at three different scales (130%, 180%, and 230% of its original dimension) of a micro-CT of trabecular bone, donated by the School of Mechanical & Design Engineering at the University of Portsmouth, the UK (see Supplementary Materials Figure S3). It should be noted that 100% scaling could not be printed with proper fidelity due to the intrinsic resolution limits of the technique and the printer.

The printing of the scaffolds was carried out with the fused deposition technique using the Prusa i3 3D printer described on Section 2.3, a printing temperature of 195 °C, a layer thickness of 0.1 mm, a fill density of 100%, a rectilinear infill pattern, a 45 mm/s perimeter print speed, and an 80 mm/s infill print speed.

### 2.5. Cell Adhesion and Proliferation in the Scaffolds

To determine the metabolic activity and cell proliferation, the cells were seeded at 2.5 × 10^6^ cells per scaffold, left to adhere for about 30 min, and then placed in 24-well plates and incubated at 37 °C and in 5% CO_2_ for 1, 7, and 30 days. As controls, scaffolds without cells and wells without scaffolds were incubated in the same conditions. Metabolic activity was measured using Alamar Blue (Invitrogen/Thermo Fisher Scientific, Waltham, MA, USA) according to the manufacturer’s instructions at days 1, 7, and 30 after seeding the cells onto the scaffolds. Briefly, a 10% Alamar Blue solution was prepared in cell culture medium. The samples were transferred into this solution and incubated for 1 h at 37 °C and 5% CO_2_. Then, the fluorescence was measured at 544/590–10 nm (Ex/Em) in a Fluostar Optima plate reader (Ortenberg, Germany). The samples were measured in triplicate. Then, the scaffolds were washed with PBS 1X, Karnovsky-fixed, dehydrated with graded ethanol solutions, and then air-dried and sputtered with gold to a 10 nm thickness (EMS 150R ES). A current of 20 mA was used for the scanning electron microscopy analysis (Hitachi TM-3000, Tokyo, Japan).

To observe the cell adhesion in the scaffolds, a polydimethylsiloxane (PDMS) flow cell was fabricated. The PDMS was made with 10% (*m*/*m*) curing agent and 90% (*m*/*m*) elastomer base (Dow SYLGARD™ 184 Elastomer Kit, Midland, MI, USA) and mixed in a fume hood for 8 min. The mixture was placed in a vacuum chamber to remove air bubbles. The PDMS was then poured into the mold and cured at 70 °C for 2 h. The PDMS cell was cut, and oxygen plasma treatment was performed with a PDC-32G Plasma Cleaner (Harrick Plasma, Ithaca, NY, USA) to adhere the PDMS parts to coverslips. The scaffold was placed in the middle, and a syringe system was used for input and output. A total of 2.5 × 10^6^ cells per well were seeded and incubated at 37 °C for 10, 15, 20, and 25 h. For imaging, the cells were stained with 1:2000 Hoechst 33342 (Invitrogen/Thermo Fisher Scientific, Waltham, MA, USA), the cell growth was observed in an LP fluorescence microscope Model DMi8 (Leica, Wetzlar, Germany) with a blue DAPI filter (Leica, Exc: 350/50 nm; DC: 400 nm; Em: 460/50 nm), and the results were recorded using a scientific Zyla 4.2 PLUS sCMOS camera (Andor Technology/Oxford Instruments, Concord, MA, USA).

### 2.6. Statistical Analysis

Statistical analysis was performed to determine the effect of the materials on both viability and cytotoxicity using R version 4.4.1 (15 June 2024) [41], with N = 12 replicates per condition. The data were analyzed by weighted least squares ANOVA, followed by Tukey’s post hoc test to compare all possible pairs of conditions. A global *p*-value < 0.05 was considered significant. In the statistical box plot comparison, outlier points were included in the calculation of the whiskers following the 1.5 interquartile range (1.5 IQR) method.

## 3. Results

### 3.1. Viability and Cytotoxicity Assays by Extracts

**Figure 1:**
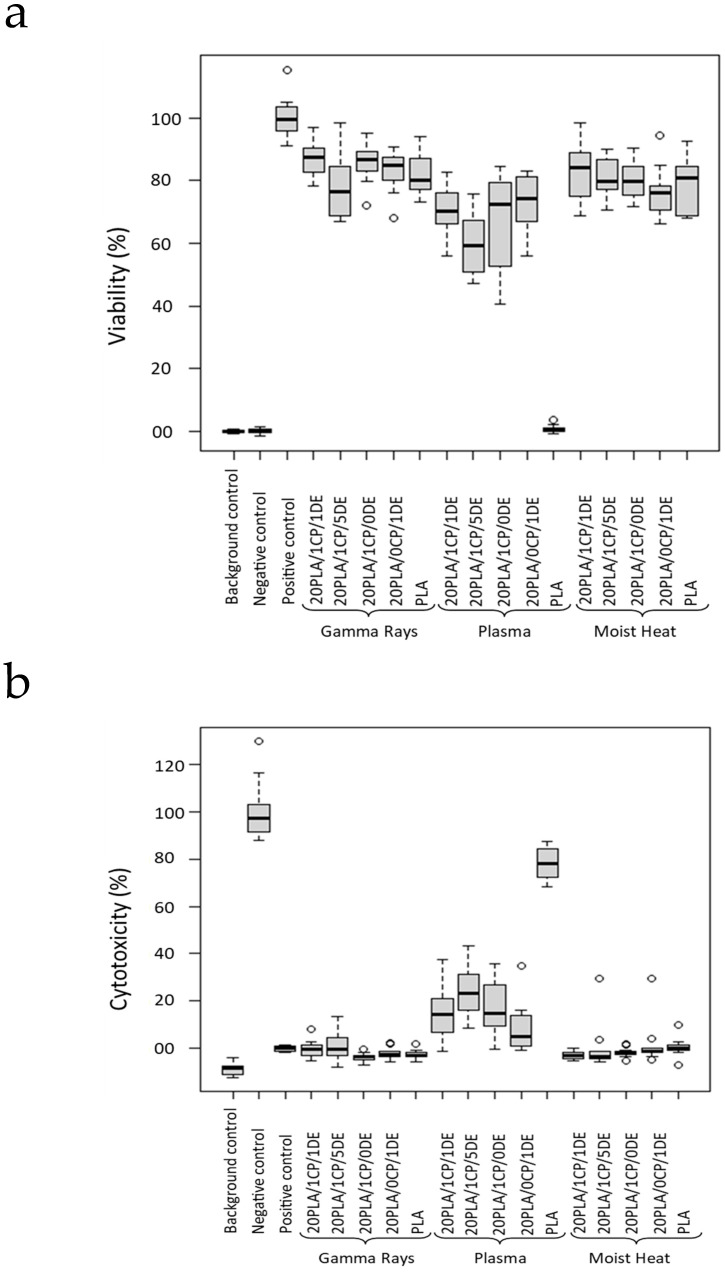
Viability of the cells (**a**) and cytotoxicity of the composites (**b**) when using three different sterilization methods. Comparison was made when sterilizing the scaffolds with gamma irradiation (25 kGy), moist heat (121 °C at 15 pounds for 15 min), or O_2_ gas plasma. *p* < 0.05.

The sterilization process is known to affect the chemical and mechanical properties of a material [42] which may also modify its biocompatibility. As shown in Figure 1, the cell viability varies among the different sterilization methods, and it also varies among the different composites. 

**Figure 2:**
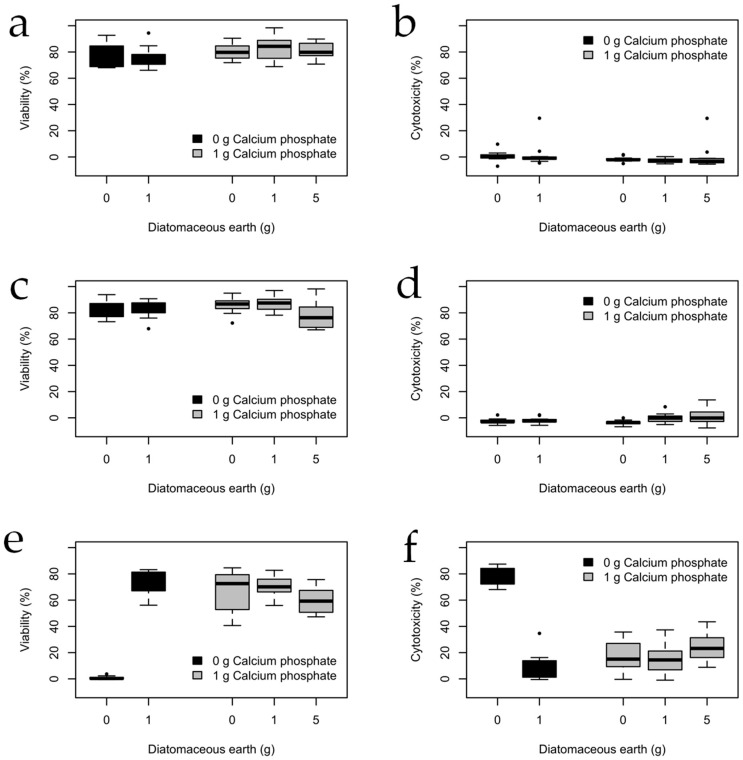
Viability and cytotoxicity of the MC3T3 cells cultured in scaffolds made of composites containing 20 g of PLA sterilized by (**a**,**b**) moist heat, (**c**,**d**) gamma rays, and (**e**,**f**) plasma sterilization. Viability was determined by the MTT test, and cytotoxicity was estimated by quantifying the release of the LDH enzyme. (N_samples_ = 12 for each condition).

The next step was to determine the effect of sterilization on PLA composites with different additive ratios, as shown in Figure 2. The results are discussed in Section 4.

### 3.2. Mechanical and Thermal Characterization

Given that the future goal would be to incorporate these composites as scaffolds into patients, a sterilization technique compatible with the composites is needed. Gamma irradiation has the advantage of killing microorganisms and inactivating viruses without a significant rise in the temperature of the irradiated material [43], which is important given the relatively low T_g_ of the PLA used (*ca*. 60 °C in our case). Supplementary Materials Figure S2 shows the sterilized 3D-printed probes made of the composites. There is no visual difference before and after gamma irradiation.

Degradation tests with the gamma-treated 3D-printed probes were performed for up to 13 weeks in buffered media at a pH of 7.4 and at 37 °C with stirring. The masses of the probes were measured before and after, and no significant loss resulted, except for the composite with a high loading of DE (20 PLA/1CP/5DE), which experienced a 1.3% mass loss. This composite was also the only one that caused a significant change in the pH of the buffered solution (the final pH was 6.85; the other solutions in contact with the probes had pHs no lower than 7.33 on average). This shows that most of the composites show the short-term stability required for proper scaffolding before subsequent biodegradation. The degradation of PLA inside the body has been found to be dependent on the molecular weight, chirality, and crystallinity of the PLA, as well as the local pH and temperature within the body, with reports as low as 40 weeks and as high as over 6 years [5].

**Figure 3:**
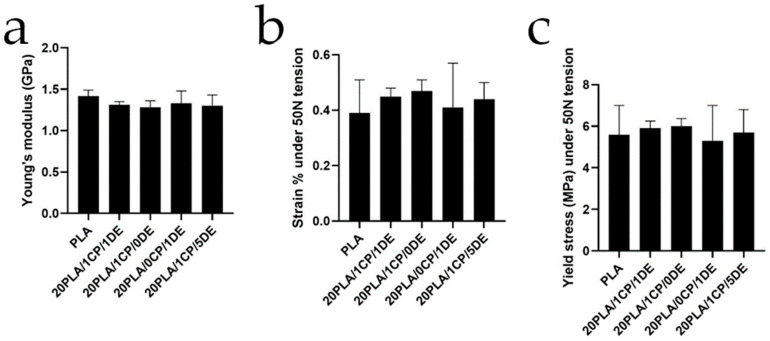
Mechanical properties in tension analysis for gamma-irradiated 3D-printed probes after 13 weeks of stirring under simulated physiological conditions. (**a**) Young’s modulus, (**b**) strain, and (**c**) yield stress. Error bars shown are standard deviation. No significant differences were found in the studied materials.

Figure 3 summarizes the findings from the tension analysis performed for each gamma-sterilized composite after 13 weeks under simulated physiological conditions. Their thermal properties are summarized in Table 4.

**Figure 4:**
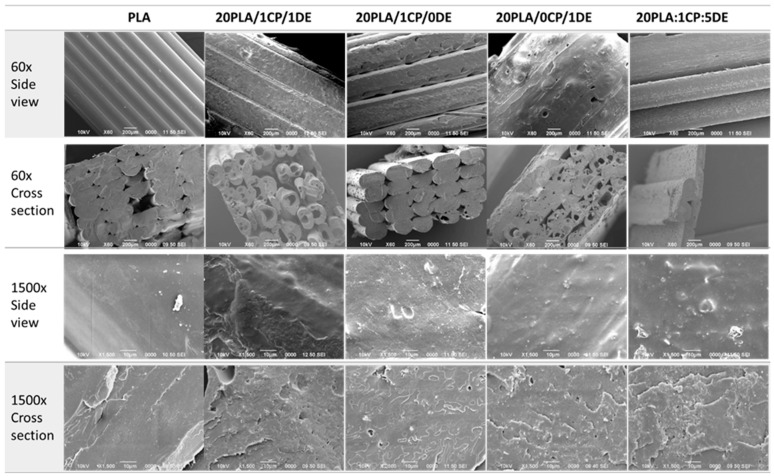
SEM images at 60× and 1500× magnification of 3D-printed probes with different compositions. Scale bars are 200 μm for 60× and 10 μm for 1500× magnifications.

Figure 4 shows 60× and 1500× magnifications of the 3D-printed composites in side views and cross-section views (with cryo-fracture). It is evident that the pure PLA sample prints better, as observed in the low-magnification side views. The DE additive increases the porosity, as observed in the low-magnification cross-sections, which is not observed when only CP is used. At high magnifications, both the exterior and the cross-sections of the composites with additives show some roughness compared to pure PLA.

### 3.3. Metabolic Activity and Cell Adhesion in Scaffolds

**Figure 5:**
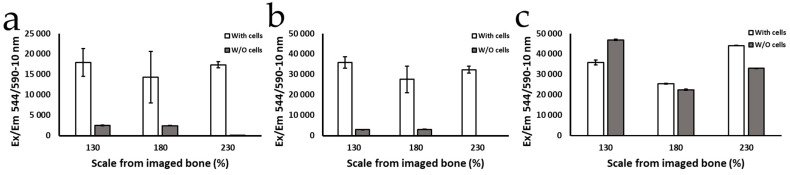
Metabolic activity of MC-3T3 cells growing on the 20PLA/1CP/1DE scaffold after (**a**) 1 day, (**b**) 7 days, and (**c**) 30 days determined by the Alamar Blue assay. The scale of bone % refers to the percentage of the printing magnification relative to the size of spongy bone revealed by a micro-CT scan.

In order to determine which porosity was the most biocompatible, the metabolic activity and cell adhesion of the MC3T3-E1 cell line were determined through the use of Alamar Blue reagent (Figure 5).

**Figure 6:**
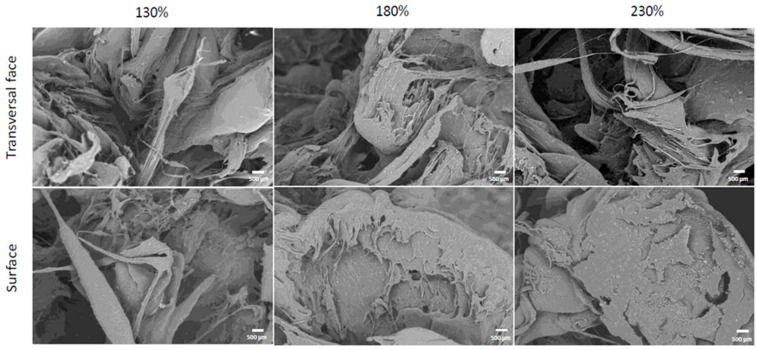
SEM images of MC-3T3 cells (bright, grain-like features) adhered to the composite 20PLA/1CP/1DE scaffold after 7 days. Scale bars represent 500 μm.

The results of the analysis carried out through SEM to visualize cell adhesion are shown in Figure 6, which shows cells adhered to the transverse and superficial faces of the scaffolds of each porosity; the scaffolds are covered with cells, some of which have a more typical morphology similar to that observed in vitro, while in other areas, the cells are closer together, and they show variable morphologies.

## 4. Discussion

Plasma sterilization is a widely used method, although oxygen plasma sterilization can induce surface modifications, which include zeta-potential increases [44] and hydrophilicity increases [45], in part due to the emergence of new oxygen-rich species on the surface and a rise in surface roughness [46]. In our study, we observed a reduction in cell viability of the cells exposed to the extract of the plasma-treated composites (Figure 1), likely due to the aforementioned reactive oxygen species generated in the process.

For composites sterilized by moist heat and gamma radiation, the cell viability results determined by the MTT test were similar and close to the those for the positive control, which is in accordance with what has been described before, that is, that gamma rays and moist heat do not increase the cytotoxicity level of PLA [47,48,49]. However, the samples treated with moist heat showed slight deformation, which was likely related to the low T_g_ of PLA, and some displayed a change in color. Neijhoft et al. recently concluded that autoclaving while maintaining the dimensional stability in 3D-printed PLA is only possible for diameters above 5 mm [50].

With moist heat and gamma rays (Figure 2a–d), there is no significant difference in variability as the level of DE is increased (or whether CP is present or not); however, for plasma sterilization (Figure 2e,f), there is a significant difference between pure PLA and the composites: the viability is close to 0% for the O_2_-plasma-treated pure PLA (cytotoxicity *ca*. 80%), and the plasma-treated composites have a lower viability (a higher cytotoxicity) than the composites treated with the other sterilization treatments.

PLA is one of the most commonly used polymers in tissue engineering. It degrades to form lactic acid, which is common in the human body, but can decrease the pH of the medium and negatively affect the surrounding cells [51]. In the case of 1 g of calcium phosphate without DE, the viability is much higher than that of pure PLA, which might be related to calcium phosphate acting as a buffer and its reported benefits for osteoimplants [52], as it promotes cell adhesion, proliferation, the production of osteoblastic differentiation markers, and bone formation [53].

When 1 g of DE is added, the viability of PLA with and without calcium phosphate increases. Silica in DE promotes the proliferation and differentiation of osteoblasts, as well as collagen synthesis, among other benefits [18,19]. For example, in conjunction with calcium phosphate, it enhances the differentiation of osteoblasts [29,31]. Nevertheless, a small (and not significant) decrease in cell viability is observed when 5 g of DE is used. Silicon’s effects on osteoblasts are seen at concentrations up to 100 ppm, while on osteoclasts, these effects are dose-dependent, with stimulation at low concentrations (below 30 ppm) and inhibition at higher levels [27]. Low silicon concentrations on implant surfaces promote osseous remodeling, while higher concentrations enhance tissue differentiation [54].

As observed in Figure 3, all the composites have similar behavior up to our instrument’s tension limit of 50 N. Their Young’s moduli compare favorably to that of cancellous bone (0.442 GPa) [55], which makes these composites mechanically acceptable as scaffolding implants. As shown in Table 4, the melting points do not change significantly with the additives, which allowed all of them to be extruded at 175 °C and 3D-printed at 195 °C. The crystallinity, calculated using the second heating enthalpy of fusion [56], is maintained for all the composites relative to that of pure PLA. A T_g_ value of 59 °C is well below the temperature used in moist heat sterilization, which explains the deformation and whitening observed for the samples treated with that sterilization method.

The results of the Alamar Blue tests (Figure 5) show that during the first 24 h, as well as the first 7 days, the cells growing onto the scaffolds were viable, and the population increased with no statistically significant differences among the evaluated porosities. However, after 30 days of culture, the metabolic activity detected in the scaffolds with 130% porosity scaling turned out to be higher than in that in the others with greater scaling. As described by Chen et al. and Zhang et al. for other materials, the difference in cell viability between scaffolds could be due to the fact that the scaffolds with 130% porosity magnification showed pores with a better morphology, as well as a larger surface area [57,58]. These characteristics provide a more ideal environment for cell proliferation that might be closer to physiologic conditions than scaffolds with larger pores. 

Despite the difference in the porosity of the scaffolds, all three evaluated scaffolds show that the material used to fabricate them is suitable for cell adhesion and proliferation, as suggested by the measured fluorescence intensity throughout the assay, indicating that the cells proliferated and remained metabolically active, growing on the scaffolds for at least 30 days, in line with results obtained by others [59,60]. This suggests that the biomaterial presents characteristics associated with appropriate cell biocompatibility.

The cell adhesion, displayed as bright grain-like features in the scanning electron microscopy images in Figure 6, is similar to the results presented in the literature [58,59,61,62]. Despite their variable morphology, the cells were able to adhere correctly externally, and there were cells present inside the scaffolds; therefore, they colonized not only the outside of the scaffolds but the interior surfaces as well. These results are promising, as they indicate that the material promotes osteoconduction, one of the main properties necessary for osseointegration [63,64].

The best cell adhesion was found at the 130% printing magnification, followed by 180%, using a quantitative analysis of the SEM images. Even though the 130% and 180% printing magnifications showed cells at a greater quantity than at the 230% scale, there was no notable difference in morphology between the porosities, corresponding to the results described by Luan et al. [65] 

Additionally, this was confirmed using fluorescence micrographs taken of the 20PLA/1CP/1DE scaffold material inside the PDMS flow cell that was incubated with the MC-3T3 cells (see Supplementary Materials Figure S4). After 10 h of incubation, it was possible to observe cells attached to the composite scaffold and a population of cells on the surface of the scaffold. Although the imaged regions and morphologies of the scaffold changed, qualitative differences between the populations at 10 h and 25 h were observed. 

Therefore, considering the MTT tests, the Alamar Blue assays, the SEM analyses, and the fluorescence microscopy images, the biomaterial developed in this study is considered biocompatible, presenting good cell adhesion, proliferation, viability, and osteoconduction.

## 5. Conclusions

It was demonstrated that it is possible to generate biomimetic grafts or scaffolds for bone regeneration based on a composite material made of polylactic acid (PLA) and different percentages of silica (SiO_2_), obtained from frustules of ocean diatoms, and 3D-printed based on computerized axial tomography of trabecular bone.

The viability of the cells decreased when all the composites were sterilized by plasma. Since moist heat deformed the test pieces, sterilization with gamma rays is considered optimal for these composites. There was no significant difference in the mechanical properties measured for the gamma-sterilized probes after 13 weeks of degradation conditions, and their Young’s modulus values were above those of cancellous bone. There is no significant difference in PLA’s crystallinity or other thermal properties when PLA is mixed with DE and CP at the amounts used here. The DE additive adds micro-porosity to the printed samples when compared with pure PLA.

In addition, it is shown that the cell adhesion and proliferation were similar at all scales of the scaffold, except at 30 days, where the scaffolds printed at a scale of 130% presented the highest cell population, as indicated by the highest metabolic activity. Finally, the 130% scale scaffold provides evidence that the scaffolds permit osteoconduction.

Future studies, including a gene expression profile of the cells growing on the scaffolds printed with the selected customized materials, are recommended. Special attention should be devoted to sequences related to osteogenic differentiation genes, such as ALP, COL1A1, SPP1, BGLAP, osteocalcin, osteopontin, and Runx2. Moreover, animal and clinical studies to substantiate the use of 3D-printed silica-filled composites as an alternative to homologous implants for various bone regeneration applications are also envisioned.

## Figures and Tables

**Table 1:** Material properties and supplier summary.

Material	Supplier	Properties
Polylactic Acid (PLA 3D850)	NatureWorks/SmartMaterials (Plymouth, MN, USA)	Density: 1.24 g/cm³ Tensile Strength: 57.7 MPa (XY) Flexion Strength: 103.6 MPa (XY) Hardness: 84.4 Shore D Glass Transition Temp: 65 °C
Calcium Phosphate (β-Tricalcium phosphate 21218)	Sigma-Aldrich/Merck (St. Louis, MO, USA)	Molecular Weight: 310.18 g/mol Density: 3.14 g/cm³ Water Solubility: 7.7 g/L
Diatomaceous Earth (Costalite NS Agro)	Sur Química (San José, Costa Rica)	Density: 0.3–0.5 g/cm³ SiO_2_ Content: 85% CaO Content: 0.30% pH: 4–5.5 Porosity: High
Chloroform for Analysis EMSURE^®^ 102445	Sigma-Aldrich/Merck (St. Louis, MO, USA)	Molecular Weight: 119.38 g/mol Density: 1.49 g/cm³ Water Solubility: 8.7 g/L

**Table 2:** Composite mass ratios.

Composite	PLA	Calcium Phosphate (CP)	Diatomaceous Earth (DE)
PLA	100	-	-
20PLA/1CP/1DE	20	1	1
20PLA/1CP/0DE	20	1	0
20PLA/0CP/1DE	20	0	1
20PLA/1CP/5DE	20	1	5

**Table 3:** Sterilization methods and conditions.

Sterilization Method	Details
Moist Heat	Temperature: 121 °C Pressure: 15 psi Duration: 15 min
Gamma Radiation	Source: Co-60 Dose: 25 kGy Equipment: Ob-Servo Ignis
Oxygen Plasma	Flow Rate: 1.0 sccm O_2_ Pressure: 2.6 Torr Duration: 1.5 min Equipment: Harrick Plasma PDC-32G

**Table 4:** Glass transition temperature (T_g_), crystallization temperature (T_c_), melting temperature (T_m_) and cold crystallization temperature (T_cc_), and percent crystallinity of gamma-sterilized probes via DSC.

Composite	T_g_ (°C)	T_c_ (°C)	ΔH_c_ (J/g)	T_m1_ (°C)	ΔH_m1_ (J/g)	T_m2_ (°C)	ΔH_m2_ (J/g)	T_cc_ (°C)	ΔH_cc_ (J/g)	PLA % Crystallinity
PLA	59.4	95.7	24.7	176.8	51.0	175.5	48.0	107.5	22.7	51.2
20PLA/1CP/1DE	56.2	88.0	23.3	177.6	56.2	171.3/177.6	40.4	113.2	40.7	47.4
20PLA/1CP/0DE	56.9	88.1	28.5	175.7	63.1	175.8	48.8	108.1	41.3	54.7
20PLA/0CP/1DE	58.3	86.4	23.4	175.8	61.3	170.5/176.9	43.4	113.6	43.3	48.6
20PLA/1CP/5DE	58.7	93.1	23.2	175.9	49.5	176.8	36.4	111.4	32.8	50.5

## Data Availability

The original contributions presented in this study are included in the article/Supplementary Material; further inquiries can be directed to the corresponding author.

## Supplementary Materials

The following supporting information can be downloaded at https://www.mdpi.com/article/10.3390/bioengineering11111059/s1. SEM characterization of diatomaceous earth, pictures of 3D prints for mechanical testing, and fluorescence microscopy of MC-3T3 cells adhered to composite 3D-printed scaffold within a fluidic cell. Figure S1: SEM images of DE at various positions and magnifications displaying a variety of particle sizes and morphologies; Figure S2: The 3D-printed and gamma-sterilized composite probes; Table S1. Mechanical properties of the composites, after gamma sterilization, and after gamma + 13 weeks under simulated physiological conditions in PBS. Figure S3: Composite filaments and 3D-printed scaffolds. Figure S4. Adhesion of MC-3T3 cells onto the composite 20PLA/1CP/1DE scaffolds.
