# Supplementary material for: Composites of Polylactic Acid with Diatomaceous Earth for 3D-Printing Biocompatible Scaffolds: A Systematic Study of Their Mechanical, Thermal, and Biocompatibility Properties
Source: Bioengineering (Basel). 2024 Oct 24;11(11):1059. doi: 10.3390/bioengineering11111059 (PMC11591056; doi:10.3390/bioengineering11111059)
Supplement: Supplementary file 1 [file bioengineering-11-01059-s001.zip › bioengineering-3108739-supplementary.pdf]

Supplementary Material

Composites of polylactic acid with diatomaceous earth for 3D printing biocompatible scaffolds: systematic study of the mechanical, thermal and biocompatible properties

Authors: Lilliam Trejos-Soto[1,2], Gabriel O. Rivas-Hernández [2,4†], Rodrigo Mora-Bolaños [3,5†], Nathalia Vargas-Valverde [3,6†], Abraham Valerio [7,8†], Andrea Ulloa-Fernández [2], Jorge Oviedo-Quirós [10,11], Alfonso García-Piñeres [12], Sergio A. Paniagua[3], Carolina Centeno-Cerdas [2,9] and Leonardo Lesser-Rojas [7,13,*]

[1] Master Program of Engineering in Medical Devices, School of Materials Science and Engineering, Tecnológico de Costa Rica, Cartago 30101, Costa Rica
[2] Biotechnology Research Center (CIB), Biology School, Tecnológico de Costa Rica, Cartago 30101, Costa Rica., ccenteno@itcr.ac.cr
[3] National Nanotechnology Laboratory (LANOTEC), National Center for High Technology (CENAT), San José 1174, Costa Rica.; spaniagua@cenat.ac.cr
[4] Bioengineering Department, Universidad Carlos III de Madrid, Leganés Madrid 28911, Spain
[5] Advanced Materials Science and Engineering Master Degree (AMASE), Université de Lorraine, Nancy 54000, France
[6] Faculty of Chemistry and Biology, Université Grenoble Alpes, Saint Martin d'Hères 38400, France.
[7] School of Physics, Universidad de Costa Rica, San José 11501, Costa Rica
[8] Advanced Materials and Liquid Crystal Institute & Materials Science Graduate Program, Kent State University, Kent, OH 44242, USA
[9] Department of Biochemistry, School of Medicine, Universidad de Costa Rica, San José 11501, Costa Rica.
[10] Craniomaxillofacial Cleft Palate Unit, National Children's Hospital "Dr. Carlos Sáenz Herrera", San José 10103, Costa Rica
[11] Faculty of Dentistry, Universidad de Costa Rica, San José 11501, Costa Rica.
[12] Cellular and Molecular Biology Research Center (CIBCM), Universidad de Costa Rica, San José 11501, Costa Rica.
[13] Research Center in Atomic, Nuclear and Molecular Sciences (CICANUM), Universidad de Costa Rica, San José 11501, Costa Rica

1) Authors to whom correspondence should be addressed: leonardo.lesser@ucr.ac.cr. **Fax:** (+506) 2253-7017. **Postal address:** Apartado Postal 11501-2060, San Pedro de Montes de Oca, San José, Costa Rica

†) Current affi liation

Section I

Characterization of diatomaceous earth

Figure 1 shows SEM images of the DE used. EDX on multiple spots found on average 60.0±3% oxygen, 38.0±3% silicon and 1.8±0.8% aluminum by weight, with no carbon present. As observed, most of the particles are ~10 microns in size.

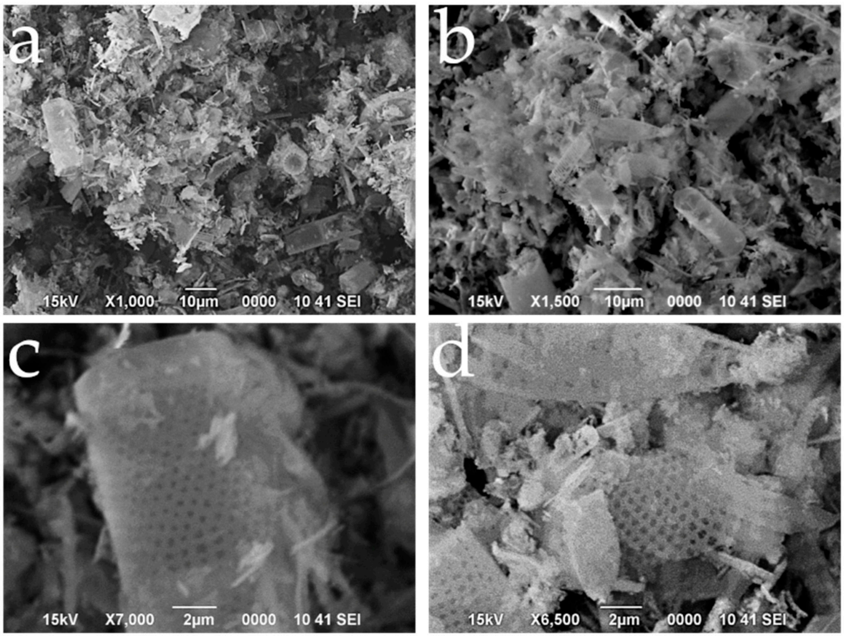

Figure S1. SEM images of DE at various positions and magnifications displaying a variety of particle sizes and morphology. Scale bars are presented in each picture and represent 10 microns in (**a**) and (**b**), and 2 microns in (**c**) and (**d**).

Section II

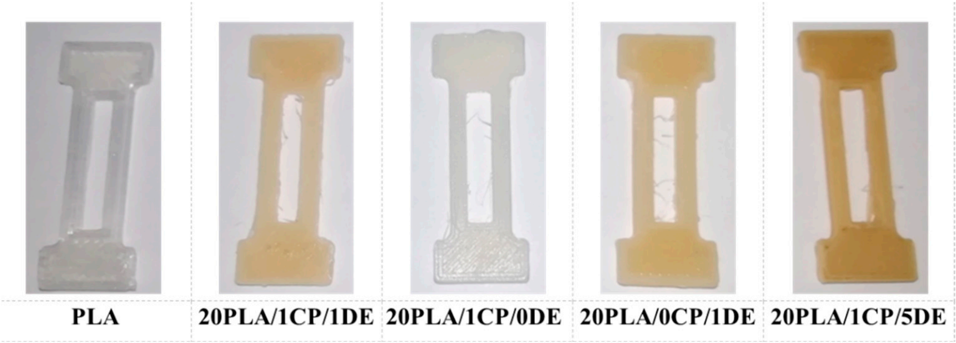

Figure S2. 3D-printed and Gamma-sterilized composite probes. The scaffolds, are made of three chemical components in five different proportions which are stated below the pictures.

Table S1. Mechanical properties of the composites, after gamma sterilization and after gamma + 13 weeks under simulated physiological conditions in PBS.

	Young´s modulus (GPa)	*Std. Dev. Young´s Modulus (GPa)*	*Strain %*	*Std. Dev. Strain %*	*Yield Stress (MPa)*	*Std. Dev. Yield Stress (MPa)*
PLA	0.91	0.09	0.67	0.07	6.0	0.3
PLA gamma	0.60	0.03	0.40	0.14	2.37	0.66
PLA gamma 13 week PBS	1.42	0.07	0.39	0.12	5.60	1.40
20PLA/1CP/1DE	1.06	0.12	0.62	0.06	6.56	0.56
20PLA/1CP/1DE gamma	0.70	0.06	0.58	0.22	4.0	1.3
20PLA/1CP/1DE gamma 13 week PBS	1.31	0.04	0.45	0.03	5.91	0.34
20PLA/1CP	1.18	0.08	0.48	0.06	5.64	0.64
20PLA/1CP gamma	0.74	0.08	0.7	0.08	5.1	0.2
20PLA/1CP gamma 13 week PBS	1.28	0.08	0.47	0.04	6.01	0.36
20PLA/1DE	1.47	0.21	0.43	0.01	6.26	0.89
20PLA/1DE gamma	0.56	0.03	0.59	0.2	3.3	1.3
20PLA/1DE gamma 13 week PBS	1.33	0.15	0.41	0.16	5.3	1.7
20PLA/1CP/5DE	1.41	0.11	0.39	0.04	5.43	0.38
20PLA/1CP/5DE gamma	0.64	0.24	0.37	0.06	2.3	0.5
20PLA/1CP/5DE gamma 13 week PBS	1.30	0.13	0.44	0.06	5.7	1.1

Section III

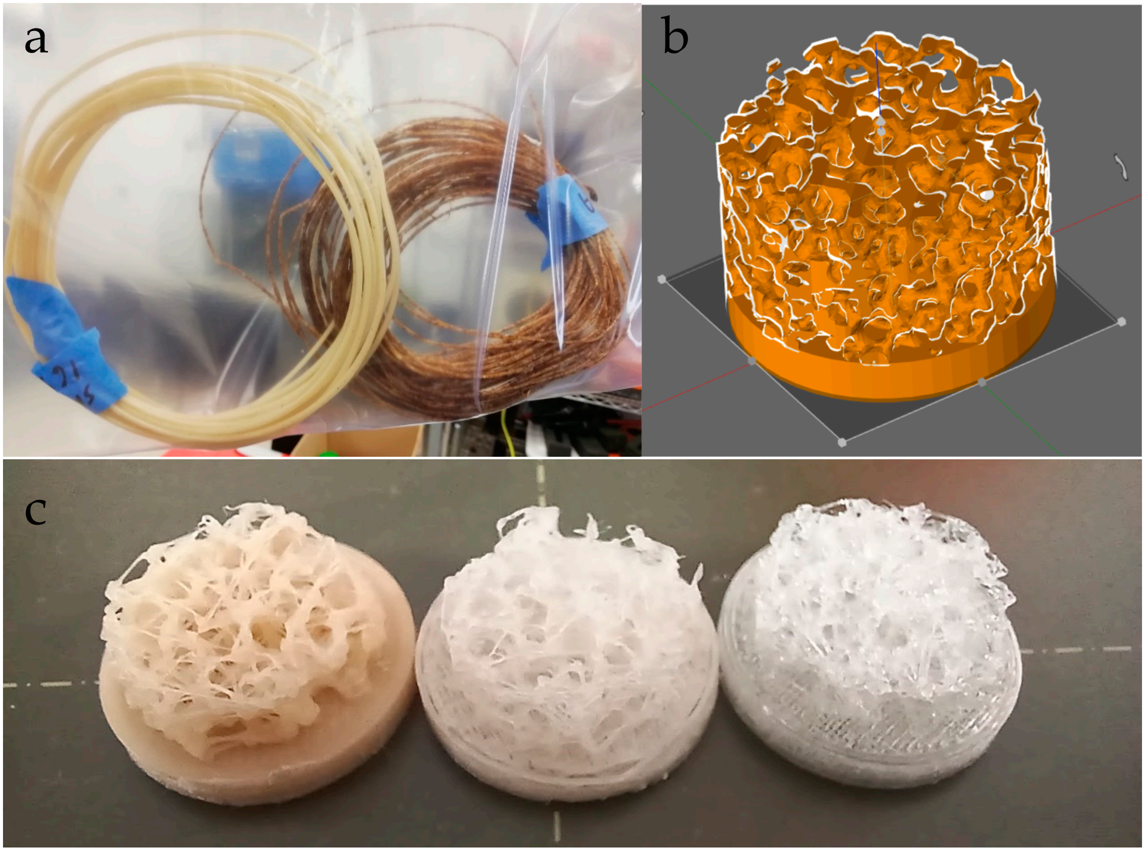

Figure S3. Composite filaments and 3D-printed scaffolds. (**a**) Extruded filaments, (**b**) Micro-CT model of trabecular bone converted to an *.STL file and imported to the 3D printing software, (**c**) 3D printed scaffolds of 20PLA/1CP/1DE, 20PLA/1CP/0DE and control with only PLA (left to right). Scale is 130% of a Micro-CT of trabecular bone dimensions. Diameter of the base in (**b**) and (**c**) is ~10 mm.

Section IV

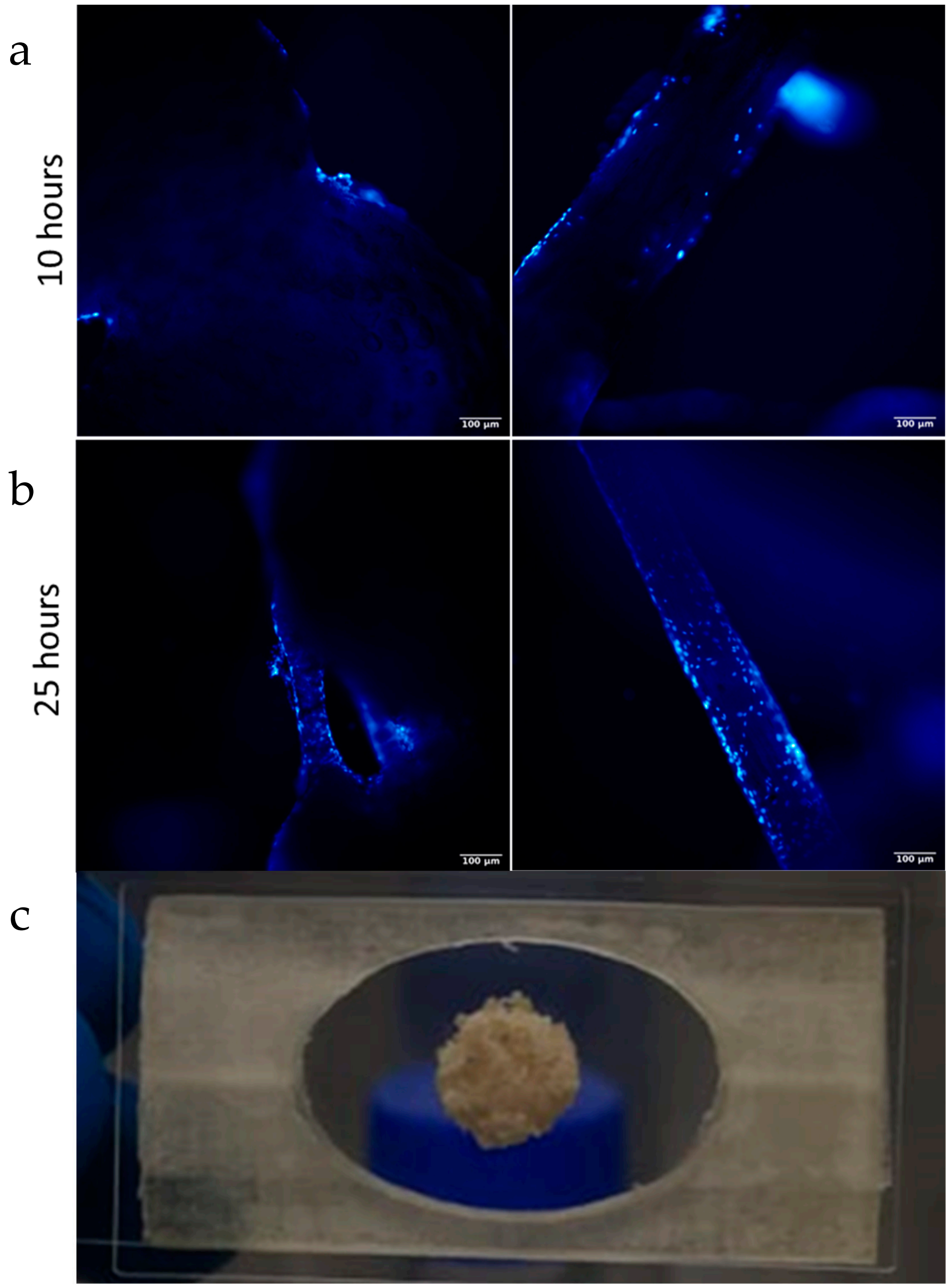

Figure S4. Adhesion of MC-3T3 cells onto the composite 20PLA/1CP/1DE scaffolds after (**a**) 10 h and (**b**) 25 h of incubation inside a fluidic cell (**c**). The cells were stained with Hoescht 33342. Scale bars represent 100 µm.
